# Use of Labor Neuraxial Analgesia for Vaginal Delivery and Severe Maternal Morbidity

**DOI:** 10.1001/jamanetworkopen.2022.0137

**Published:** 2022-02-22

**Authors:** Jean Guglielminotti, Ruth Landau, Jamie Daw, Alexander M. Friedman, Stanford Chihuri, Guohua Li

**Affiliations:** 1 Department of Anesthesiology, Columbia University Vagelos College of Physicians and Surgeons, New York, New York; 2 Department of Health Policy and Management, Columbia University Mailman School of Public Health, New York, New York; 3 Division of Maternal Fetal Medicine, Department of Obstetrics and Gynecology, Columbia University Vagelos College of Physicians and Surgeons, New York, New York; 4 Department of Epidemiology, Columbia University Mailman School of Public Health, New York, New York

## Abstract

**Importance:**

Addressing severe maternal morbidity (SMM) is a public health priority in the US. Use of labor neuraxial analgesia for vaginal delivery is suggested to reduce the risk of postpartum hemorrhage (PPH), the leading cause of preventable severe maternal morbidity.

**Objective:**

To assess the association between the use of labor neuraxial analgesia for vaginal delivery and SMM.

**Design, Setting, and Participants:**

In this population-based cross-sectional study, women aged 15 to 49 years undergoing their first vaginal delivery were included. Data were taken from hospital discharge records from New York between January 2010 and December 2017. Data were analyzed from November 2020 to November 2021.

**Exposures:**

Neuraxial analgesia (ie, epidural or combined spinal-epidural) vs no neuraxial analgesia.

**Main Outcomes and Measures:**

The primary outcome was SMM, as defined by the US Centers for Disease Control and Prevention, and the secondary outcome was PPH. Adjusted odds ratios (aORs) and 95% CIs of SMM associated with neuraxial analgesia were estimated using the inverse propensity score–weighting method and stratified according to race and ethnicity (non-Hispanic White vs racial and ethnic minority women, including non-Hispanic Asian or Pacific Islander, non-Hispanic Black, Hispanic, and other race and ethnicity) and to the comorbidity index for obstetric patients (low-risk vs high-risk women). The proportion of the association of neuraxial analgesia with the risk of SMM mediated through PPH was estimated using mediation analysis.

**Results:**

Of 575 524 included women, the mean (SD) age was 28 (6) years, and 46 065 (8.0%) were non-Hispanic Asian or Pacific Islander, 88 577 (15.4%) were non-Hispanic Black, 104 866 (18.2%) were Hispanic, 258 276 (44.9%) were non-Hispanic White, and 74 534 (13.0%) were other race and ethnicity. A total of 400 346 women (69.6%) were in the low-risk group and 175 178 (30.4%) in the high-risk group, and 272 921 women (47.4%) received neuraxial analgesia. SMM occurred in 7712 women (1.3%), of which 2748 (35.6%) had PPH. Before weighting, the incidence of SMM was 1.3% (3486 of 272 291) with neuraxial analgesia compared with 1.4% (4226 of 302 603) without neuraxial analgesia (risk difference, −0.12 per 100; 95% CI, −0.17 to −0.07). After weighting, the aOR of SMM associated with neuraxial analgesia was 0.86 (95% CI, 0.82-0.90). Decreased risk of SMM associated with neuraxial analgesia was similar between non-Hispanic White women and racial and ethnic minority women and between low-risk and high-risk women. More than one-fifth (21%; 95% CI, 14-28) of the observed association of neuraxial analgesia with the risk of SMM was mediated through the decreased risk of PPH.

**Conclusions and Relevance:**

Findings from this study suggest that use of neuraxial analgesia for vaginal delivery is associated with a 14% decrease in the risk of SMM. Increasing access to and utilization of labor neuraxial analgesia may contribute to improving maternal health outcomes.

## Key Points

**Question:**

Is use of labor neuraxial analgesia for vaginal delivery associated with decreased risk of severe maternal morbidity?

**Findings:**

In this cross-sectional study of 575 524 women with vaginal delivery in New York hospitals from 2010 to 2017, use of neuraxial analgesia was associated with a 14% decrease in risk of severe maternal morbidity.

**Meaning:**

Increasing the access to and utilization of labor neuraxial analgesia may help reduce severe maternal morbidity and improve maternal health outcomes.

## Teaser

This cross-sectional study assesses the association of use of labor neuraxial analgesia for vaginal delivery with severe maternal morbidity.

## Introduction

In 2020, addressing severe maternal morbidity (SMM) was defined as a public health priority by the US Department of Health and Human Services.^1,2^ Indeed, the reported incidence of SMM has more than doubled between 1999 and 2017, affecting approximately 1 in 60 women in 2017.^3^ Of concern, the risk of SMM is up to 3-fold increased for racial and ethnic minority women compared with non-Hispanic White women.^4,5,6,7^ As of 2021, postpartum hemorrhage (PPH) remains the leading cause of preventable SMM and maternal mortality.^8,9,10,11^

Labor neuraxial analgesia (ie, epidural or combined spinal-epidural analgesia) is the most effective technique to alleviate labor pain and is used in 70% of birthing women in the US.^12,13^ Use of neuraxial analgesia has been associated with reduced risk of severe PPH. One study analyzing vaginal births between 2004 and 2006 in France reported a 47% decreased risk of severe PPH, defined as a decrease in postpartum hemoglobin concentration greater than 4 g/dL (to convert to grams per liter, multiply by 10), for women who received labor neuraxial analgesia compared with those who did not.^14^ The proposed mechanism is that the presence of the epidural catheter allows immediate and effective management of PPH because interventions to stop the bleeding require anesthesia (eg, manual removal of a retained placenta). Timely management of obstetric hemorrhage after a vaginal delivery prevents escalation into severe hemorrhage, with potentially superimposed coagulation defects (eg, disseminated intravascular coagulation). Replication of the association identified in the French study is important, given advances in obstetric and anesthesia care practices since the study was conducted, and the marked differences in the health care systems and maternal health outcomes between the US and France.^15,16,17,18^ For instance, the maternal mortality ratio in the US is twice the maternal mortality ratio in France and other high-income countries.^16,17^ Using data from a large cohort of vaginal deliveries in New York hospitals, the objective of this study was to assess the association between labor neuraxial analgesia and SMM.

## Methods

The study protocol was granted exemption under 45 Code of Federal Regulation 46 (not human subjects research) by the Institutional Review Board of Columbia University Irving Medical Center. This study followed the Strengthening the Reporting of Observational Studies in Epidemiology (STROBE) reporting guideline.

### Data System

Data for this study came from the New York State Inpatient Database (SID), collected as part of the Healthcare Cost and Utilization Project (HCUP) sponsored by the Agency for Healthcare Research and Quality.^19^ The SID includes patient characteristics, diagnoses, and procedure codes for all inpatient discharges from nonfederal acute care community hospitals. This analysis was limited to New York, as it is the only Healthcare Cost and Utilization Project–participating state also providing information on anesthesia care.^20,21^

### Study Sample

The study sample included hospitalizations for vaginal delivery among women aged 15 to 49 years between January 1, 2010, and December 31, 2017. Vaginal delivery cases were identified using previously published algorithms.^22,23^ If a woman had more than 1 delivery during the study period, only the first was included. Women were excluded if (1) information on anesthesia care was missing or did not correspond to no neuraxial analgesia or to neuraxial analgesia, (2) the hospital American Hospital Association identifier or hospital Federal Information Processing Standards county code were missing, (3) the hospital had no labor and delivery unit, and (4) the hospital had less than 10 annual deliveries. Women requiring general anesthesia for urgent cesarean delivery after neuraxial analgesia was placed were excluded.

### Exposure

The exposure of interest was labor neuraxial analgesia (ie, epidural or combined spinal-epidural) compared with no neuraxial analgesia. In SID data, anesthesia care is reported as a categorical variable with values corresponding to no anesthesia care, local anesthesia, general anesthesia, regional anesthesia (ie, neuraxial), other anesthesia, and missing. For the purpose of the study, the study sample was limited to discharges recording no anesthesia care or regional (neuraxial) analgesia. Because of the high proportion of discharges excluded for missing information on anesthesia care (189 825 of 950 649 [19.9%] hospitalizations for first vaginal delivery), we conducted a sensitivity analysis including these discharges and handling missing values for anesthesia care with multiple imputation. The comparison of women excluded because of missing information on anesthesia care and women included in the study sample is presented in eTable 1 in the Supplement.

### Outcome

The primary outcome was SMM according to the US Centers for Disease Control and Prevention definition, which includes 16 maternal complications (eg, heart failure) and 5 procedures (eg, hysterectomy).^24^ The Centers for Disease Control and Prevention definition has a sensitivity of 77% and specificity of 99%, using individual medical record analysis as the criterion standard.^25^ The secondary outcome was PPH (*International Classification of Diseases, Ninth Revision, Clinical Modification *[*ICD*-*9*-*CM*] diagnosis codes 666.0 to 666.2 and *ICD*-*10*-*CM* codes O72.0 to O72.2).

### Women and Hospital Characteristics

Women characteristics were recorded directly from the SID, including age (19 years and younger, 20 to 29 years, 30 to 39 years, and 40 years and older), race and ethnicity, residence (rural or urban), health insurance (Medicaid, Medicare, private, self-pay, or other), and admission day (weekday or weekend). In the SID, race and ethnicity includes 7 mutually exclusive categories: non-Hispanic Asian or Pacific Islander, non-Hispanic Black, Hispanic, Native American, non-Hispanic White, other race and ethnicity, and missing. Because of the low number of Native American women in the study sample, these women were included in the other race and ethnicity group. Individual comorbidities were summarized using the comorbidity index for obstetric patients (CMI-OB; categorized into low risk [CMI-OB = 0] vs high risk [CMI-OB of 1 or greater]).^26,27^ This index includes maternal age and 20 maternal conditions (eg, severe preeclampsia) that are predictive of maternal end-organ injury or death during the delivery hospitalization through 30 days postpartum. Possible contraindications to neuraxial analgesia and obstetrical characteristics were identified using *ICD*-*9*-*CM* and *ICD*-*10* codes (eTable 2 in the Supplement).

For each hospital, the following characteristics were calculated for each year of the study period among all deliveries using SID data: volume of delivery, cesarean delivery rate, proportion of induced labor, proportion of racial and ethnic minority women, proportion of women who are safety net patients (Medicaid or Medicare beneficiaries and uninsured), proportion of women with an CMI-OB score of 1 or greater, proportion of admissions during a weekend, proportion of neuraxial analgesic or anesthetic techniques in deliveries, and coding intensity (mean number of diagnosis and procedure codes per discharge). The number of labor and delivery units in the hospital, hospital location (rural or urban), and teaching status were abstracted from the American Hospital Association Annual Survey Database.^28^ The numbers of obstetrician and gynecologists, physician anesthesiologists, and certified registered nurse anesthetist per 1000 in-hospital births were abstracted at the hospital county level from the Area Health Resources Files.^29^

### Statistical Analysis

Using the inverse propensity score–weighting method and mediation analysis, this retrospective cross-sectional study of vaginal delivery cases examined whether labor neuraxial analgesia was associated with decreased risk of SMM and the mediating role of PPH. Two stratified analyses were planned a priori according to (1) race and ethnicity (non-Hispanic White vs racial and ethnic minority women) and (2) the CMI-OB (low-risk women vs high-risk women).

Statistical analysis was performed with R version 3.6.2 (The R Foundation) and specific packages (*mice* for multiple imputations, *Matching* for matching, and *survival* for conditional logistic regression). Mediation analysis was performed using SAS version 9.4 (SAS Institute).^30^

#### Descriptive Statistics

Comparisons of characteristics between women with and without neuraxial analgesia used the absolute standardized mean difference (SMD), with a value greater than 10% used to define a clinically important imbalance. The incidence of SMM and PPH was calculated in women with and without neuraxial analgesia overall, according to race and ethnicity (non-Hispanic White vs racial and ethnic minority women), and according to CMI-OB value (low risk vs high risk). The risk difference was calculated as the difference in SMM or PPH incidence between women with and without neuraxial analgesia. The 95% CI for the risk difference was estimated using bootstrap with replacement (B = 2000) and the percentile method.

#### Crude and Adjusted Odds Ratio for SMM and PPH Associated With Labor Neuraxial Analgesia

Crude odds ratios (ORs) of SMM and PPH associated with labor neuraxial analgesia were estimated using univariate logistic regression models overall, according to race and ethnicity, and according to CMI-OB value. Adjusted ORs were estimated using the inverse propensity score–weighting method and stabilized weights (eFigure 1 in the Supplement). The propensity score estimated the individual probability of receiving neuraxial analgesia. It was calculated using a fixed-effect logistic regression model, with neuraxial analgesia as the dependent variable and the 37 patient-level and hospital-level characteristics listed in eTable 3 in the Supplement as the independent variables. Interaction terms and quadratic terms were included in the propensity score. A complete case analysis was performed because less than 1% of discharges (n = 4967) had missing values for variables included in the propensity score. Balance after weighting was assessed using the SMD. Adjusted ORs for SMM and PPH associated with neuraxial analgesia were estimated using weighted logistic regression models overall, according to race and ethnicity, and according to CMI-OB value and with further adjustment for variables with a persistent imbalance after weighting.

To assess the robustness of the main analysis, the following sensitivity analyses were performed. First, the adjusted OR of SMM associated with neuraxial analgesia was estimated using stabilized weights truncated at 1% and at 99%. Second, we used the propensity score–matching method. Matching used the nearest-neighbor approach with a caliper of 0.2 and 1 case matched to 1 control. Adjusted OR of SMM associated with neuraxial analgesia were estimated using conditional logistic regression models, with further adjustment for variables with persistent imbalance after matching (eTable 4 in the Supplement). Third, discharges with missing information on anesthesia care were included and handled using multiple imputation and adjusted OR of SMM associated with neuraxial analgesia estimated using the inverse propensity score–weighting method and stabilized weights, as previously described.

#### Mediation Analysis

We used the unified interaction and mediation analysis framework to decompose the association of labor neuraxial analgesia with the risk SMM into a direct association and an indirect association (eFigure 2 in the Supplement).^31^ The direct association corresponds to the association of neuraxial analgesia with the risk of SMM in the absence of PPH and the indirect association to the association of labor neuraxial analgesia with the risk of SMM mediated through PPH. The mediation analysis was performed using propensity score–matched data and variables with persistent imbalance after matching were included in the models.

#### Sample Size and Study Power

Because there is no validated solution to estimate the required sample size or study power using the inverse propensity score–weighting method, we do not provide such estimates.^32^

## Results

During the study period, 575 524 women with vaginal delivery were included and analyzed (eFigure 3 in the Supplement). The mean (SD) age was 28 (6) years, and 46 065 (8.0%) were non-Hispanic Asian or Pacific Islander, 88 577 (15.4%) were non-Hispanic Black, 104 866 (18.2%) were Hispanic, 258 276 (44.9%) were non-Hispanic White, and 74 534 (13.0%) were other race and ethnicity. A total of 400 346 women (69.6%) were in the low-risk group and 175 178 (30.4%) in the high-risk group, and 272 921 women (47.4%) received neuraxial analgesia.

Compared with women without labor neuraxial analgesia, women with labor neuraxial analgesia were more likely to be non-Hispanic White, have private health insurance, premature rupture of membranes, an induced labor, or fetal heart rhythm abnormalities (Table 1). Women with neuraxial labor analgesia were also likely to give birth in a high-volume teaching hospital, with a higher utilization of neuraxial techniques, and with a higher number of obstetrician and gynecologists, physician anesthesiologists, or nurse anesthetists.

**Table 1.** Comparison of Women Who Did Not Receive Neuraxial Analgesia for Vaginal Delivery With Those Who Did, Before and After Inverse Propensity Score Weighting (New York State Hospitals, 2010-2017)

Characteristic	Before weighting			After weighting		
	No. (%)		SMD, %	No. (%)		SMD, %
	No neuraxial analgesia (n = 302 603)	Neuraxial analgesia (n = 272 921)		No neuraxial analgesia (n = 304 734)	Neuraxial analgesia (n = 219 308)	
General characteristics						
Age, y						
≤19	21 983 (7.3)	17 594 (6.4)	7.3	20 346 (6.7)	14 347 (6.5)	1.8
20-29	153 824 (50.8)	131 552 (48.2)		151 343 (49.7)	107 307 (48.9)	
30-39	117 305 (38.8)	115 127 (42.2)		123 094 (40.4)	90 385 (41.2)	
≥40	9491 (3.1)	8648 (3.2)		9951 (3.3)	7269 (3.3)	
Race^a^						
Non-Hispanic Asian or Pacific Islander	20 796 (6.9)	25 269 (9.3)	30.8	23 308 (7.6)	18 366 (8.4)	9.0
Non-Hispanic Black	54 749 (18.2)	33 828 (12.4)		46 650 (15.3)	29 350 (13.4)	
Hispanic	65 924 (22.0)	38 942 (14.3)		56 460 (18.5)	36 476 (16.6)	
Non-Hispanic White	118 533 (39.5)	139 743 (51.4)		135 300 (44.4)	105 061 (47.9)	
Other	40 219 (13.4)	34 315 (12.6)		43 016 (14.1)	30 055 (13.7)	
Missing, No.	2382	824		0	0	
Residence						
Rural	19 261 (6.4)	12 517 (4.6)	7.9	16 976 (5.6)	12 695 (5.8)	<0.1
Urban	282 252 (93.3)	259 733 (95.2)		287 758 (94.4)	206 613 (94.2)	
Missing, No.	1090	671		0	0	
Insurance type						
Medicaid	172 351 (57.0)	111 516 (40.9)	39.0	150 560 (49.4)	104 071 (47.5)	7.5
Medicare	1382 (0.5)	1101 (0.4)		1284 (0.4)	1035 (0.5)	
Private	109 611 (36.2)	149 732 (54.9)		137 696 (45.2)	104 559 (47.7)	
Self-pay (uninsured)	11 354 (3.8)	4494 (1.6)		8071 (2.6)	3956 (1.8)	
Other	7902 (2.6)	6078 (2.2)		7123 (2.3)	5687 (2.6)	
Missing, No.	NA^b^	0		0	0	
CMI-OB						
0 (Low risk)	214 471 (70.9)	185 875 (68.1)	6.0	211 159 (69.3)	150 698 (68.7)	1.2
≥1 (High risk)	88 132 (29.1)	87 046 (31.9)		93 575 (30.7)	68 610 (31.3)	
Obesity	15 349 (5.1)	13 579 (5.0)	<1.0	16 451 (5.4)	10 299 (4.7)	3.2
Possible contraindications to neuraxial anesthesia						
Coagulation factor deficit, Von Willebrand disease, and thrombocytopenia	5757 (1.9)	6107 (2.2)	2.4	6645 (2.2)	4796 (2.2)	<0.1
Fever or infection during labor	2412 (0.8)	2634 (1.0)	1.8	2825 (0.9)	1826 (0.8)	1.0
Chorioamnionitis	6044 (2.0)	7622 (2.8)	5.2	7576 (2.5)	4830 (2.2)	1.9
Pregnancy and labor characteristics						
Admission for delivery during a weekend	72 604 (24.0)	64 144 (23.5)	1.2	72 460 (23.8)	52 128 (23.8)	<0.1
Pregnancy resulting from ART	553 (0.2)	1148 (0.4)	4.2	1046 (0.3)	755 (0.3)	<0.1
Previous cesarean delivery	8257 (2.7)	7476 (2.7)	<1.0	8513 (2.8)	6171 (2.8)	<0.1
Uterus fibroid	2392 (0.8)	2485 (0.9)	1.3	3090 (1.0)	1901 (0.9)	1.5
Polyhydramnios	1911 (0.6)	2308 (0.8)	2.5	2409 (0.8)	1664 (0.8)	<0.1
Placenta praevia	401 (0.1)	360 (0.1)	<0.1	443 (0.1)	291 (0.1)	<0.1
Placenta accreta	913 (0.3)	1171 (0.4)	2.1	1179 (0.4)	864 (0.4)	<0.1
Multiple gestation	2003 (0.7)	2495 (0.9)	2.9	2728 (0.9)	1782 (0.8)	<0.1
Abnormal presentation	8999 (3.0)	8062 (3.0)	<1.0	9815 (3.2)	6723 (3.1)	<0.1
Preterm delivery	17 077 (5.6)	12 705 (4.7)	4.5	15 848 (5.2)	12 237 (5.6)	1.7
Premature rupture of membranes	17 461 (5.8)	24 265 (8.9)	12.0	22 116 (7.3)	16 444 (7.5)	<0.1
Induction of labor	56 458 (18.7)	67 178 (24.6)	14.5	70 891 (23.3)	47 901 (21.8)	3.4
Abnormal fetal heart rhythm	38 509 (12.7)	50 405 (18.5)	15.9	51 328 (16.8)	34 198 (15.6)	3.4
Hospital characteristics						
Teaching hospital	240 277 (79.4)	229 617 (84.1)	12.3	251 094 (82.4)	176 903 (80.7)	4.5
Rural hospital	16 330 (5.4)	7461 (2.7)	13.5	12 475 (4.1)	8792 (4.0)	<0.1
Annual deliveries, mean (SD)	2684 (1797)	3455 (2137)	39.1	3181 (2185)	3224 (2065)	2.0
Cesarean delivery rate, mean (SD)	33.3 (6.5)	34.4 (6.5)	16.7	33.7 (6.5)	33.8 (6.5)	2.1
Proportion of induction of labor, mean (SD)	16.4 (7.6)	18.3 (7.8)	24.9	17.7 (7.6)	17.6 (7.6)	1.7
Proportion of racial and ethnic minority women, mean (SD)	57.9 (31.2)	48.7 (26.3)	32.2	53.8 (29.3)	50.5 (28.2)	11.5
Proportion of women who are safety net patients, mean (SD)^c^	58.2 (27.5)	43.5 (24.2)	56.8	51.4 (26.5)	47.8 (24.9)	13.8
Proportion of women with CMI-OB ≥1, mean (SD)	43.0 (6.5)	45.3 (7.1)	33.8	44.5 (7.1)	44.3 (7.5)	3.7
Proportion of admission for delivery during a weekend, mean (SD)	20.7 (2.0)	20.5 (2.2)	7.4	20.6 (2.1)	20.7 (2.1)	3.9
Proportion of neuraxial techniques in deliveries, mean (SD)	22.1 (28.0)	71.8 (17.3)	213.3	46.0 (33.8)	58.5 (25.6)	41.6
Coding intensity, mean (SD)	8.0 (1.5)	8.3 (1.6)	24.9	8.2 (1.6)	8.2 (1.6)	3.4
Hospital county characteristics (per 1000 in-hospital births in the county), mean (SD)						
Obstetricians and gynecologists	13.4 (4.7)	14.7 (4.7)	27.7	14.0 (4.8)	14.0 (4.9)	1.1
Physician anesthesiologists	15.5 (6.9)	17.7 (6.2)	33.8	16.4 (6.8)	16.6 (6.7)	3.1
Certified registered nurse anesthetists	4.9 (4.4)	6.3 (5.5)	27.9	5.5 (5.2)	5.9 (5.3)	8.7
Year of delivery						
2010-2011	91 737 (30.3)	79 609 (29.2)	5.1	82 272 (27.0)	66 774 (30.4)	9.8
2012-2013	79 802 (26.4)	69 220 (25.4)		75 810 (24.9)	53 705 (24.5)	
2014-2015	75 845 (25.1)	69 411 (25.4)		87 131 (28.6)	54 555 (24.9)	
2016-2017	55 219 (18.2)	54 681 (20.0)		59 520 (19.5)	44 274 (20.2)	

Abbreviations: ART, assisted reproductive technology; CMI-OB, comorbidity index for obstetric patients; NA, not applicable; SMD, standardized mean difference.

^a^ Data on race were taken from the New York State Inpatient Database. In the State Inpatient Database, race and ethnicity includes 7 mutually exclusive categories: non-Hispanic Asian or Pacific Islander, non-Hispanic Black, Hispanic, Native American, non-Hispanic White, other race and ethnicity, and missing. Because of the low number of Native American women in the study sample, these women were included in the other race and ethnicity group.

^b^ Because of Healthcare Cost and Utilization Project data use agreement restrictions on small cell size, the number of observed cases and exact proportions are not presented.

^c^ Safety net patients are Medicaid beneficiaries, Medicare beneficiaries, and uninsured.

### Crude OR for SMM and PPH Associated With Labor Neuraxial Analgesia

SMM occurred in 7712 women (1.3%; 95% CI, 1.31-1.37), of which 2748 (35.6%) had PPH. Before weighting, the incidence of SMM among women with labor neuraxial analgesia was 1.3% (3486 of 272 291) vs 1.4% (4226 of 302 603) among women without, yielding a risk difference of −0.12% (95% CI, −0.17 to −0.07) and a crude OR of 0.91 (95% CI, 0.87-0.96) (Table 2). Conversely, the incidence of PPH was significantly higher in women with neuraxial analgesia compared with women without neuraxial analgesia (Table 2).

**Table 2.** Crude ORs of Severe Maternal Morbidity and Postpartum Hemorrhage Associated With Neuraxial Analgesia for Vaginal Delivery Before Inverse Propensity Score Weighting (New York Hospitals, 2010-2017)

Variable	No neuraxial analgesia			Neuraxial analgesia			Risk difference, % (95% CI)^a^	Crude OR (95% CI)
	Women, No.	Events, No.	Incidence, % (95% CI)	Women, No.	Events, No.	Incidence, % (95% CI)		
All women								
SMM	302 603	4226	1.40 (1.36 to 1.44)	272 921	3486	1.28 (1.23 to 1.32)	−0.12 (−0.17 to −0.07)	0.91 (0.87 to 0.96)
PPH	302 603	9889	3.27 (3.20 to 3.33)	272 921	9269	3.40 (3.33 to 3.46)	0.13 (0.06 to 0.21)	1.04 (1.01 to 1.07)
Non-Hispanic White women								
SMM	118 533	1129	0.95 (0.90 to 1.01)	139 743	1412	1.01 (0.96 to 1.06)	0.06 (−0.01 to 0.12)	1.06 (0.98 to 1.15)
PPH	118 533	3623	3.06 (2.96 to 3.17)	139 743	4389	3.14 (3.05 to 3.23)	0.08 (−0.03 to 0.19)	1.03 (0.98 to 1.08)
Racial and ethnic minority women^b^								
SMM	181 688	3078	1.69 (1.63 to 1.75)	132 354	2067	1.56 (1.50 to 1.63)	−0.13 (−0.21 to −0.06)	0.92 (0.87 to 0.97)
PPH	181 688	6199	3.41 (3.33 to 3.50)	132 354	4866	3.68 (3.58 to 3.78)	0.27 (0.15 to 0.37)	1.08 (1.04 to 1.12)
Low-risk women (CMI-OB = 0)								
SMM	214 471	2329	1.09 (1.04 to 1.13)	185 875	1819	0.98 (0.93 to 1.02)	−0.11 (−0.16 to −0.05)	0.90 (0.85 to 0.96)
PPH	214 471	6358	2.96 (2.89 to 3.04)	185 875	5579	3.00 (2.92 to 3.08)	0.04 (−0.05 to 0.13)	1.01 (0.98 to 1.05)
High-risk women (CMI-OB ≥1)								
SMM	88 132	1897	2.15 (2.06 to 2.25)	87 046	1667	1.91 (1.82 to 2.01)	−0.24 (−0.35 to −0.13)	0.89 (0.83 to 0.95)
PPH	88 132	3531	4.01 (3.88 to 4.14)	87 046	3690	4.24 (4.11 to 4.37)	0.23 (0.08 to 0.39)	1.06 (1.01 to 1.11)

Abbreviations: CMI-OB, comorbidity index for obstetric patients; OR, odds ratio; PPH, postpartum hemorrhage; SMM, severe maternal morbidity.

^a^ 95% CI estimated using bootstrap with replacement (B = 2000) and the percentile method.

^b^ Racial and ethnic minority women included the following categories: Asian or Pacific Islander, Black, Hispanic, Native American, and other. Information on race or ethnicity was missing for 3206 hospitalizations.

### Adjusted OR for SMM and PPH Associated With Labor Neuraxial Analgesia

After weighting, the risk difference for SMM between women with and without neuraxial analgesia was −0.21% (95% CI, −0.30 to −0.12) and the adjusted OR for SMM associated with labor neuraxial analgesia 0.86 (95% CI, 0.82-0.90) (Table 3). The decreased risk of SMM was similar between non-Hispanic White women and racial and ethnic minority women and between low-risk and high-risk women. Contrary to the unadjusted analysis, labor neuraxial analgesia was associated with a decreased risk of PPH (adjusted OR, 0.91; 95% CI, 0.88-0.94). A post hoc analysis identified hospital characteristics as the factors accounting for the inversion of the OR of PPH (eTable 5 in the Supplement), particularly the hospital utilization of neuraxial techniques (eTable 6 in the Supplement).

**Table 3.** Adjusted ORs of Severe Maternal Morbidity and Postpartum Hemorrhage Associated With Neuraxial Analgesia for Vaginal Delivery After Inverse Propensity Score Weighting (New York Hospitals, 2010-2017)

Variable	No neuraxial analgesia			Neuraxial analgesia			Risk difference, % (95% CI)^a^	Adjusted OR (95% CI)	
	Women, No.	Events, No.	Incidence, % (95% CI)	Women, No.	Events, No.	Incidence, % (95% CI)		Without further adjustment^b^	With further adjustment^c^
All women									
SMM	304 734	4427	1.45 (1.38 to 1.53)	219 308	2727	1.24 (1.17 to 1.32)	−0.21 (−0.30 to −0.12)	0.85 (0.81 to 0.90)	0.86 (0.82 to 0.90)
PPH	304 734	10796	3.54 (3.42 to 3.66)	219 308	7277	3.32 (3.21 to 3.43)	−0.22 (−0.36 to −0.09)	0.93 (0.91 to 0.96)	0.91 (0.88 to 0.94)
Non-Hispanic White women									
SMM	135 300	1473	1.09 (0.98 to 1.21)	105 061	1069	1.02 (0.90 to 1.14)	−0.07 (−0.21 to 0.08)	0.93 (0.86 to 1.01)	0.92 (0.85 to 0.99)
PPH	135 300	4561	3.37 (3.19 to 3.56)	105 061	3247	3.09 (2.93 to 3.26)	−0.28 (−0.49 to −0.07)	0.91 (0.87 to 0.95)	0.89 (0.85 to 0.93)
Racial and ethnic minority women^d^									
SMM	169 434	2954	1.74 (1.64 to 1.85)	114 247	1658	1.45 (1.36 to 1.54)	−0.29 (−0.41 to −0.17)	0.83 (0.78 to 0.88)	0.82 (0.78 to 0.87)
PPH	169 434	6235	3.68 (3.53 to 3.84)	114 247	4030	3.53 (3.38 to 3.68)	−0.15 (−0.33 to −0.01)	0.96 (0.92 to 0.99)	0.93 (0.89 to 0.96)
Low-risk women (CMI-OB = 0)									
SMM	211 159	2279	1.08 (1.01 to 1.15)	150 698	1467	0.97 (0.89 to 1.06)	−0.11 (−0.20 to −0.01)	0.90 (0.85 to 0.96)	0.93 (0.87 to 0.98)
PPH	211 159	6784	3.21 (3.08 to 3.35)	150 698	4446	2.95 (2.82 to 3.09)	−0.26 (−0.42 to −0.09)	0.92 (0.88 to 0.95)	0.89 (0.86 to 0.92)
High-risk women (CMI-OB ≥1)									
SMM	93 575	2148	2.30 (2.11 to 2.50)	68 610	1261	1.84 (1.70 to 1.98)	−0.46 (−0.67 to −0.26)	0.80 (0.74 to 0.85)	0.79 (0.74 to 0.85)
PPH	93 575	4011	4.29 (4.04 to 4.54)	68 610	2830	4.12 (3.93 to 4.32)	−0.17 (−0.42 to 0.11)	0.96 (0.92 to 1.01)	0.95 (0.90 to 0.99)

Abbreviations: CMI-OB, comorbidity index for obstetric patients; OR, odds ratio; PPH, postpartum hemorrhage; SMM, severe maternal morbidity.

^a^ 95% CI estimated using bootstrap with replacement (B = 2000) and the percentile method.

^b^ OR estimated using a weighted logistic regression, without further adjustment for variables with persistent imbalance after weighting.

^c^ OR estimated using a weighted logistic regression, with further adjustment for 3 variables with persistent imbalance after weighting: (1) hospital proportion of racial and ethnic minority women, (2) hospital proportion of women who were safety net patients, and (3) hospital proportion of neuraxial analgesic or anesthetic techniques in deliveries.

^d^ Racial and ethnic minority women included the following categories: Asian or Pacific Islander, Black, Hispanic, Native American, and other.

In the sensitivity analyses (Table 4), the adjusted OR of SMM associated with labor neuraxial analgesia was 0.90 (95% CI, 0.85-0.94) with stabilized weights truncated at 1% and 99%, 0.91 (95% CI, 0.83-0.99) with the propensity score–matching method, and 0.91 (95% CI, 0.87-0.95) with handling discharges with missing information on anesthesia care using multiple imputations (Table 4).

**Table 4.** Adjusted ORs of Severe Maternal Morbidity Associated With Neuraxial Analgesia for Vaginal Delivery in the Main and Sensitivity Analyses (New York Hospitals, 2010-2017)

Analysis	No neuraxial analgesia			Neuraxial analgesia			Risk difference, % (95% CI)^a^	Adjusted OR (95% CI)
	Women, No.	Events, No.	Incidence, % (95% CI)	Women, No.	Events, No.	Incidence, % (95% CI)		
Main analysis								
Inverse propensity score weighting with stabilized weights^b^	304 734	4427	1.45 (1.38 to 1.53)	219 308	2727	1.24 (1.17 to 1.32)	−0.21 (−0.30 to −0.12)	0.86 (0.82 to 0.90)
Sensitivity analyses								
Inverse propensity score weighting with stabilized weights truncated at 1% and 99%^c^	292 572	4151	1.42 (1.38 to 1.53)	209 770	2629	1.25 (1.17 to 1.32)	−0.17 (−0.24 to −0.09)	0.90 (0.85 to 0.94)
Propensity score matching^d^	96 407	1170	1.21 (1.14 to 1.28)	96 407	1035	1.07 (1.01 to 1.14)	−0.14 (−0.22 to −0.06)	0.91 (0.83 to 0.99)
Handling of missing values for anesthesia care with multiple imputations and inverse propensity score weighting with stabilized weights^e^	382 685	5322	1.39 (1.33 to 1.45)	331 258	4087	1.23 (1.18 to 1.28)	−0.16 (−0.22 to −0.09)	0.91 (0.87 to 0.95)

Abbreviations: OR, odds ratio; SMM, severe maternal morbidity.

^a^ 95% CI estimated using bootstrap with replacement (B = 2000) and the percentile method.

^b^ OR estimated using a weighted logistic regression and further adjustment for 3 variables with persistent imbalance after weighting: (1) hospital proportion of racial and ethnic minority women, (2) hospital proportion of women who were safety net patients, and (3) hospital proportion of neuraxial analgesic or anesthetic techniques in deliveries.

^c^ OR estimated using a weighted logistic regression and further adjustment for 5 variables with persistent imbalance after weighting: (1) race and ethnicity, (2) health insurance type, (3) hospital proportion of racial and ethnic minority women, (4) hospital proportion of women who were safety net patients, and (5) hospital proportion of neuraxial analgesic or anesthetic techniques in deliveries.

^d^ OR estimated using a conditional logistic regression and further adjustment for 3 variables with persistent imbalance after matching: (1) hospital proportion of women with comorbidity index for obstetric patients of 1 or more, (2) hospital coding intensity, and (3) year of delivery. Matching used the nearest-neighbor approach with a caliper of 0.2 and 1 case matched to 1 control.

^e^ OR estimated using a weighted logistic regression. No persistent imbalance was observed after weighting.

### Mediation Analysis

Of the observed association of neuraxial analgesia with the risk of SMM, 79% (95% CI, 64-94) was ascribed to the direct association and 21% (95% CI, 14-28) to the indirect association (ie, mediated through the decreased risk of PPH).

## Discussion

In this study of vaginal delivery cases in New York hospitals, labor neuraxial analgesia was associated with a decreased risk of SMM, which was partially mediated through a decreased risk of PPH. Decreased risk of SMM was consistent across racial and ethnic groups and across risk groups.

Our study found the suggested association between labor neuraxial analgesia and decreased risk of PPH and extends it to decreasing the risk of SMM.^14^ Early evaluation and management of the third stage of labor should avoid escalation of PPH into severe PPH and may prevent the development of potentially superimposed coagulation defects, kidney failure, and SMM. However, decreased risk of PPH accounted for only 21% of the protective association of labor neuraxial analgesia with the risk of SMM, indicating that there are other mechanisms linking labor neuraxial analgesia to the decreased risk of SMM. Other possible mechanisms may include sustained intrapartum hemodynamic monitoring of parturient women with neuraxial analgesia, which enhances maternal monitoring and early detection of blood loss immediately after delivery; adequate intravenous access and fluid resuscitation; and continuous anesthesia availability and oversight of the process of labor and delivery and preparedness for acute events.^33^

Lower labor neuraxial analgesia utilization has been repeatedly reported among racial and ethnic minority, uninsured, and low-income obstetric patients.^13,34,35,36,37,38,39,40^ While approximately 80% of non-Hispanic White women receive labor neuraxial analgesia nationwide, 70% of non-Hispanic Black women receive labor neuraxial analgesia and only 65% of Hispanic women; approximately 75% of pregnant women with a health insurance receive labor neuraxial analgesia but only 50% of uninsured pregnant women do.^13^ Several interventions can be suggested to increase access and higher utilization of labor neuraxial analgesia for these patients. First, implementation of language-concordant educational programs have been associated with decreased misconceptions about neuraxial analgesia and increased neuraxial analgesia utilization among racial and ethnic minority women.^41^ These programs facilitate patient participation in medical decision-making by making the risks and benefits associated with treatment alternatives more understandable. Second, cost may represent a financial obstacle to receive labor neuraxial analgesia for low-income patients without health insurance coverage. The mean cost of a labor neuraxial analgesia in the US is approximately $2100, which corresponds to a monthly income of 150% of the Federal Poverty Level for a single pregnant woman. Labor neuraxial analgesia is one of the most frequent surprise bills for childbirth.^42^ Up to 13% of pregnant women are uninsured in the month of delivery.^43,44,45^ Federal policies aiming at increasing insurance coverage for low-income pregnant people may help remove this financial barrier. For example, the 2014 Medicaid expansion under the Affordable Care Act has decreased the proportion of uninsured pregnant people, but its effect on labor neuraxial analgesia utilization and maternal health outcomes has been insufficiently investigated.^46,47^ Last, continuous availability of an in-house obstetric anesthesia team is required to provide uninterrupted access to labor neuraxial analgesia. Continuous in-house coverage of obstetric anesthesia services is available in approximately 86% of hospitals with more than 1500 annual deliveries, 41% of hospitals with 500 to 1500 annual deliveries, and 15% of hospitals with less than 500 annual deliveries.^40^ Approximately 60% of childbirths in the US occur in hospitals with less than 1000 deliveries, suggesting that creating continuous in-house coverage of obstetric anesthesia services in low-volume and intermediate-volume hospitals could substantially increase access to and utilization of labor neuraxial analgesia.^48^

### Limitations

Our study has limitations. First, our study is observational in nature, and the associations between neuraxial analgesia and SMM are not necessarily causal. Second, we did not specifically assess the risk of SMM in each racial and ethnic minority group (eg, Black women) because of the low number of cases in some of these groups. Since the incidence of SMM and severe PPH is higher in American Indian and Black women compared with both White women and other racial and ethnic minority women,^4,7,11^ we cannot exclude a greater effect of labor neuraxial analgesia in American Indian or Black women. Third, our data do not contain detailed information on the type of neuraxial analgesia precluding the analysis of the association of the neuraxial techniques (epidural or combined spinal-epidural) with SMM.^49,50^ Fourth, we used the number of physicians and nurses at the hospital-county level as a proxy for the number of physicians and nurses at the individual hospital level because no current data system provides this information.^20^ While this approach may be accurate for counties with only 1 hospital, it may not be accurate for counties with more than 1 hospital. Fifth, we did not include women with labor neuraxial analgesia who later required an intrapartum cesarean delivery. The benefits of labor neuraxial analgesia in such context might have been a reduction in the utilization rate of general anesthesia and risks associated with avoidable general anesthesia, rather than a decreased risk of PPH.^51^ Sixth, identification of SMM cases was limited to the delivery hospitalization period and did not account for SMM occurring after discharge that account for approximately 15% of SMM cases.^52^

## Conclusions

In this study, use of labor neuraxial analgesia for vaginal delivery was associated with a decreased risk of SMM. Our findings suggest that increasing access to and higher utilization of labor neuraxial analgesia might help decrease severe maternal morbidity and improve maternal health outcomes in the US.

## Notes

Supplement. **eTable 1.** Comparison of women included in the study sample and women excluded for missing information on anesthesia care. **eTable 2.** *ICD*-*9*-*CM* and *ICD*-*10*-*CM* codes used in this study. **eTable 3.** Variables included in the propensity score. **eFigure 1.** Distribution of the stabilized weights. **eFigure 2.** The unified interaction and mediation analysis framework. **eFigure 3.** Flowchart of the study. **eTable 4.** Comparison of women who did not receive neuraxial analgesia for vaginal delivery and those who did, before and after propensity-score matching. **eTable 5.** Odds ratios of postpartum hemorrhage associated with neuraxial analgesia for vaginal delivery with the successive addition of patient and hospital characteristics in the propensity score used for weighting. **eTable 6. **Odds ratios of postpartum hemorrhage associated with neuraxial analgesia for vaginal delivery with the individual addition of each of the hospital characteristics to the propensity score used for weighting.
