# Supplementary material for: Use of Labor Neuraxial Analgesia for Vaginal Delivery and Severe Maternal Morbidity
Source: JAMA Netw Open. 2022 Feb 22;5(2):e220137. doi: 10.1001/jamanetworkopen.2022.0137 (PMC8864505; doi:10.1001/jamanetworkopen.2022.0137)
Supplement: Supplement. — eTable 1. Comparison of women included in the study sample and women excluded for missing information on anesthesia care. eTable 2. ICD-9-CM and ICD-10-CM codes used in this study. eTable 3. Variables included in the propensity score. eFigure 1. Distribution of the stabilized weights. eFigure 2. The unified interaction and mediation analysis framework. eFigure 3. Flowchart of the study. eTable 4. Comparison of women who did not receive neuraxial analgesia for vaginal delivery and those who did, before and after propensity-score matching. eTable 5. Odds ratios of postpartum hemorrhage associated with neuraxial analgesia for vaginal delivery with the successive addition of patient and hospital characteristics in the propensity score used for weighting. eTable 6. Odds ratios of postpartum hemorrhage associated with neuraxial analgesia for vaginal delivery with the individual addition of each of the hospital characteristics to the propensity score used for weighting. [file jamanetwopen-e220137-s001.pdf]

Supplemental Online Content

Guglielminotti J, Landau R, Daw J, Friedman AM, Chihuri S, Li G. Use of labor neuraxial analgesia for vaginal delivery and severe maternal morbidity. *JAMA Netw Open*. 2022;5(2):e220137.
doi:10.1001/jamanetworkopen.2022.0137

eTable 1. Comparison of women included in the study sample and women excluded for missing information on anesthesia care

eTable 2. *ICD-9-CM* and *ICD-10-CM* codes used in this study

eTable 3. Variables included in the propensity score

eFigure 1. Distribution of the stabilized weights

eFigure 2. The unified interaction and mediation analysis framework

eFigure 3. Flowchart of the study

eTable 4. Comparison of women who did not receive neuraxial analgesia for vaginal delivery and those who did, before and after propensity-score matching

eTable 5. Odds ratios of postpartum hemorrhage associated with neuraxial analgesia for vaginal delivery with the successive addition of patient and hospital characteristics in the propensity score used for weighting

eTable 6. Odds ratios of postpartum hemorrhage associated with neuraxial analgesia for vaginal delivery with the individual addition of each of the hospital characteristics to the propensity score used for weighting

This supplementary material has been provided by the authors to give readers additional information about their work.

© 2022 Guglielminotti J et al. *JAMA Network Open*.

eTable 1. Comparison of women included in the study sample and women excluded for missing information on anesthesia care (New York State hospitals, 2010-2017)

189,825 women had missing information on anesthesia care but 13,665 had another exclusion criteria leading to 176,160 women excluded only because of missing information on anesthesia care.

	**Study sample (n = 575,524)**	**Missing information on anesthesia care (n = 176,160)**	**SMD**
General characteristics			
Age (year)			***16.3%***
≤ 19	39,577 (6.9%)	8919 (5.1%)	
20-29	285,376 (49.6%)	76,891 (43.6%)	
30-39	232,432 (40.4%)	83,021 (47.1%)	
≥ 40	18,139 (3.2%)	7329 (4.2%)	
Race			***15.6%***
Non-Hispanic White	258,276 (45.1%)	74,274 (42.2%)	
Non-Hispanic Black	88,577 (15.5%)	25,062 (14.2%)	
Hispanic	104,866 (18.3%)	28,147 (16.0%)	
Non-Hispanic Asian or Pacific Islander	46,065 (8.0%)	19,611 (11.1%)	
Other	74,534 (13.0%)	28,976 (16.5%)	
Rural residence	31,778 (5.5%)	12,210 (6.9%)	5.8%
Insurance type			***22.4%***
Medicaid	283,867 (49.3%)	75,644 (42.9%)	
Medicare	2483 (0.4%)	629 (0.4%)	
Private	259,343 (45.1%)	95,877 (54.4%)	
Self-pay (uninsured)	15,848 (2.8%)	1944 (1.1%)	
Other	13,980 (2.4%)	2044 (1.2%)	
CMI-OB ≥ 1	175,178 (30.4%)	58,870 (33.4%)	6.4%
Obesity	28,928 (5.0%)	8993 (5.1%)	<0.1%

© 2022 Guglielminotti J et al. *JAMA Network Open*.

eTable 1 (continued)

	**Study sample (n = 575,524)**	**Missing information on anesthesia care (n = 176,160)**	**SMD**
Possible contraindications to neuraxial anesthesia			
Coagulation factor deficit, Von Willebrand disease, and thrombocytopenia	11,864 (2.1%)	3721 (2.1%)	<0.1%
Fever or infection during labor	5046 (0.9%)	1920 (1.1%)	2.2%
Chorioamnionitis	13,666 (2.4%)	3491 (2.0%)	2.7%
Pregnancy and labor characteristics			
Admission for delivery during a weekend	136,748 (23.8%)	42,366 (24.0%)	<0.1%
Pregnancy resulting from ART	1701 (0.3%)	416 (0.2%)	1.2%
Previous cesarean delivery	15,733 (2.7%)	4154 (2.4%)	2.4%
Uterus fibroid	4877 (0.8%)	1866 (1.1%)	2.2%
Polyhydramnios	4219 (0.7%)	1324 (0.8%)	<0.1%
Placenta praevia	761 (0.1%)	280 (0.2%)	<0.1%
Placenta accreta	2084 (0.4%)	377 (0.2%)	2.8%
Multiple gestation	4498 (0.8%)	1230 (0.7%)	1.0%
Abnormal presentation	17,061 (3.0%)	4105 (2.3%)	4.0%
Preterm delivery	29,782 (5.2%)	8597 (4.9%)	1.3%
Premature rupture of membranes	41,726 (7.3%)	14,467 (8.2%)	3.6%
Induction of labor	123,636 (21.5%)	27,562 (15.6%)	***15.1%***
Abnormal fetal heart rhythm	88,914 (15.4%)	27,709 (15.7%)	<0.1%

© 2022 Guglielminotti J et al. *JAMA Network Open*.

eTable 1 (continued)

	**Study sample (n = 575,524)**	**Missing information on anesthesia care (n = 176,160)**	**SMD**
Hospital characteristics			
Teaching hospital	469,894 (81.6%)	154,794 (87.9%)	***17.4%***
Rural hospital	23,791 (4.1%)	10,693 (6.1%)	8.8%
Annual number of deliveries	3050 (2003)	3804 (2043)	***37.3%***
Cesarean delivery rate	33.8% (6.5)	32.9% (5.4)	***16.4%***
Proportion of induction of labor	17.3% (7.7)	14.8% (7.9)	***32.7%***
Proportion of racial and ethnic minority women	53.5% (29.4)	56.8% (23.8)	***12.2%***
Proportion of safety net women (**a**)	51.2% (27.0)	44.1% (23.4)	***28.1%***
Proportion of women with CMI-OB ≥ 1	44.1% (6.9)	46.1% (7.2)	***28.2%***
Proportion of admission for delivery during a weekend	20.6% (2.1)	21.2% (1.8)	***29.2%***
Proportion of neuraxial analgesic or Anesthetic techniques in deliveries	45.7% (34.2)	46.2% (39.6)	1.4%
Coding intensity	8.1 (1.6)	8.3 (1.6)	7.4%
Hospital county characteristics (per 1000 in-hospital births in the county)			
Number of obstetricians and gynecologists	14.1 (4.7)	14.7 (4.0)	***14.6%***
Number of physician anesthesiologists	16.5 (6.7)	17.0 (6.1)	7.5%
Number of certified registered nurse anesthetists	5.6 (5.0)	4.9 (3.2)	***14.7%***
Other			
Year of delivery			***35.2%***
2010-2011	171,346 (29.8%)	42,351 (24.0%)	
2012-2013	149,022 (25.9%)	37,401 (21.2%)	
2014-2015	145,256 (25.2%)	35,709 (20.3%)	
2016-2017	109,900 (19.1%)	60,699 (34.5%)	

Abbreviations: ART: assisted reproductive technology; CMI-OB: comorbidity index for obstetric patients; SMD: standardized mean difference.
Footnote: Results expressed as count (%) or mean (1 standard deviation).
(**a**) Safety net women are Medicaid beneficiaries, Medicare beneficiaries, and uninsured

© 2022 Guglielminotti J et al. *JAMA Network Open*.

eTable 2. *ICD-9-CM* and *ICD-10-CM* codes used in this study

	ICD-9-CM	ICD-10-CM
Women characteristics		
Obesity	278.0, 649.1, V85.3, V85.4	E66, O99.21, Z68.3, Z68.4
Possible contraindications to neuraxial anesthesia		
Coagulation factor deficit, Von Willebrand disease, and thrombocytopenia	286, 287	D65-D69
Fever or infection during labor	659.2, 659.3	O75.2, O75.3
Chorioamnionitis	658.4	O41.1
Labor characteristics		
Pregnancy resulting from assisted reproductive technology	V23.85	O09.819
Previous cesarean delivery	654.2	O34.21
Uterus fibroid	218	D25
Polyhydramnios	657.0	O40
Placenta praevia	641.0, 641.1	O44
Placenta accreta	667.0	O43.2
Multiple gestation	V27.2-V27.7, 651	O30, O31, Z37.2-Z37.7, Z37.9
Abnormal presentation	652	O32
Preterm delivery	644.2	O60.1
Premature rupture of membranes	658.1	O42.0
Induction of labor	73.01, 73.1, 73.4 (proc.)	3E033VJ, 3E0P7GC, 0U7C7ZZ (proc.)
Abnormal fetal heart rhythm	763.81-763.83, 659.7	O76, O77.8

Abbreviations: proc.: procedure code.

© 2022 Guglielminotti J et al. *JAMA Network Open*.

eTable 3. Variables included in the propensity score

Variables included in the propensity score to estimate the individual probability of receiving neuraxial analgesia. The propensity score was estimated using a fixed-effect logistic regression with neuraxial analgesia as the dependent variable and the 37 variables listed in the table below as the independent variables. A complete case analysis was performed with 4967 discharges excluded (0.9%).

General characteristics		*Coding of continuous variables*
1	Age	As continuous
2	Race (missing = 3206)	--
3	Insurance type (missing = 3)	--
	Interactions between age, race, and insurance	
4	Rural residence (missing = 1761)	--
5	Comorbidity index for obstetric patients	As continuous
6	Obesity	--
Possible contraindications to neuraxial anesthesia		
7	Coagulation factor deficit, Von Willebrand disease, and thrombocytopenia	--
8	Fever or infection during labor	--
9	Chorioamnionitis	--
Labor and delivery characteristics		
10	Admission during a weekend	--
11	Pregnancy resulting from assisted reproductive technology	--
12	Previous cesarean delivery	--
13	Uterus fibroid	--
14	Polyhydramnios	--
15	Placenta praevia	--
16	Placenta accreta	--
17	Multiple gestation	--
18	Abnormal presentation	--
19	Preterm delivery	--
20	Premature rupture of membranes	--
21	Induction of labor	--
22	Abnormal fetal heart rhythm	--
Hospital characteristics		
23	Teaching hospital	--
24	Rural hospital	--
25	Annual number of deliveries	As quadratic
	Interactions between teaching hospital, rural hospital, and annual number of deliveries	
26	Cesarean delivery rate	As continuous
27	Proportion of induced of labor	As continuous
28	Proportion of racial and ethnic minority women	As quadratic
29	Proportion of safety net women	As quadratic
30	Proportion of women with comorbidity index for obstetric patients ≥ 1	As quadratic
31	Proportion of admission for delivery during a weekend	As continuous
32	Proportion of neuraxial analgesic or anesthetic techniques in deliveries	As quadratic
33	Coding intensity	As quadratic
Hospital county characteristics (per 1000 in-hospital births)		
34	Number of obstetricians and gynecologists	As continuous
35	Number of physician anesthesiologists	As continuous
36	Number of certified registered nurse anesthetist	As continuous
Other		
37	Year of delivery	As continuous

© 2022 Guglielminotti J et al. *JAMA Network Open*.

eFigure 1. Distribution of the stabilized weights

Distribution of the stabilized weights in women who did not receive neuraxial analgesia for vaginal delivery and those who did (New York State hospitals, 2010-2017).

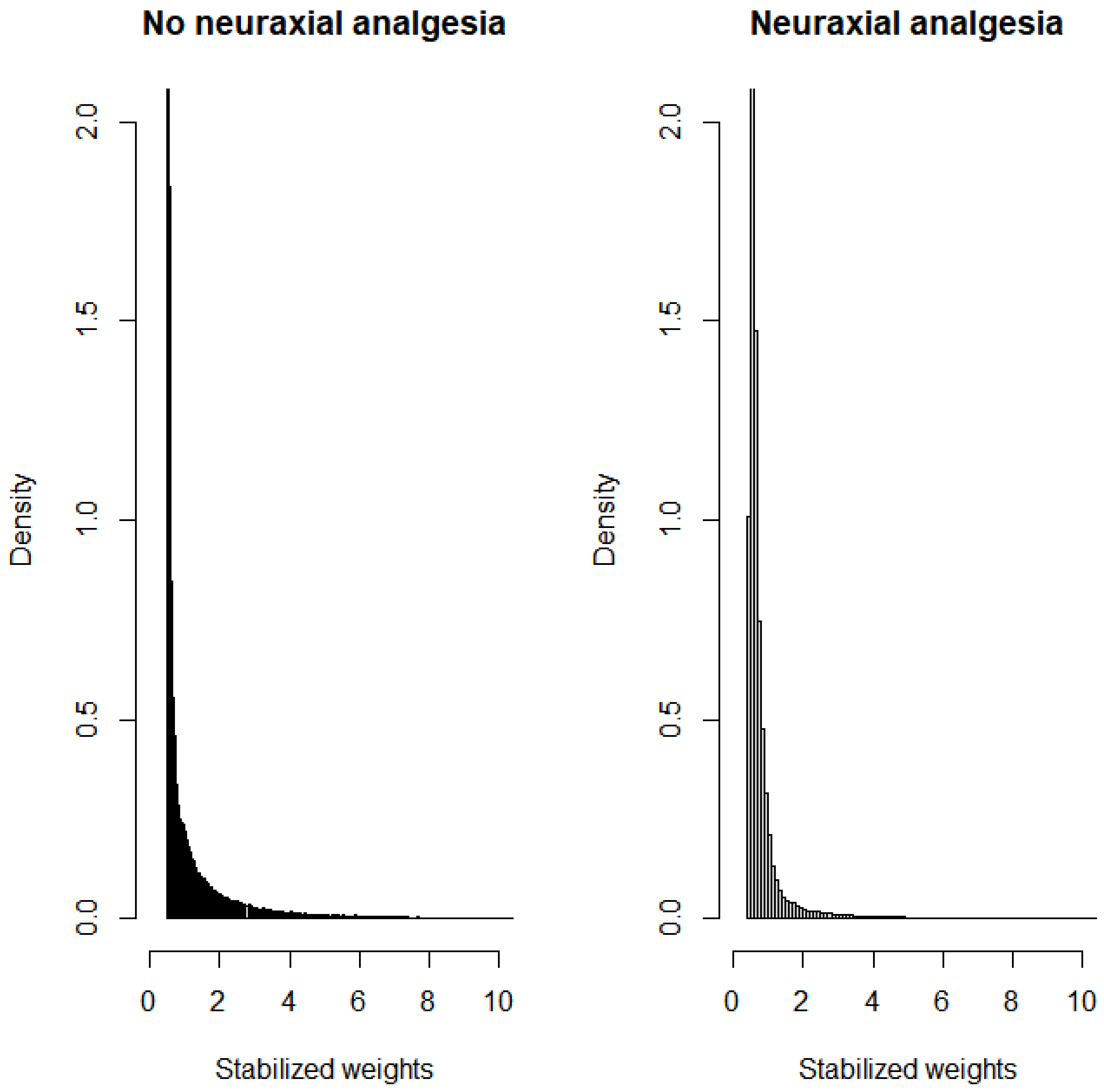

© 2022 Guglielminotti J et al. *JAMA Network Open*.

eFigure 2. The unified interaction and mediation analysis framework (VanderWeele TJ '*A unification of mediation and interaction: a 4-way decomposition.*' Epidemiology 2014;25(5): 749-761)

The total effect of labor neuraxial analgesia on severe maternal morbidity (SMM) in the presence of post-partum hemorrhage (PPH) is decomposed into 4 components: controlled direct effect, reference interaction effect, mediated interaction effect, and pure indirect effect. The sum of controlled direct effect and reference interaction effect is referred to as the **direct effect**, and the sum of mediated interaction effect and pure indirect effect as the **indirect effect**. The **controlled direct effect** describes the effect of neuraxial analgesia on SMM that is due to neither mediation through nor interaction with PPH. The **reference interaction effect** describes the effect of neuraxial analgesia that is attributable to interaction with PPH but not mediation through PPH. The **mediated interaction effect** describes the effect of neuraxial analgesia that is due to both mediation through and interaction with PPH. The **pure indirect effect** describes the effect of neuraxial analgesia that is attributable to mediation through PPH but not interaction with PPH.

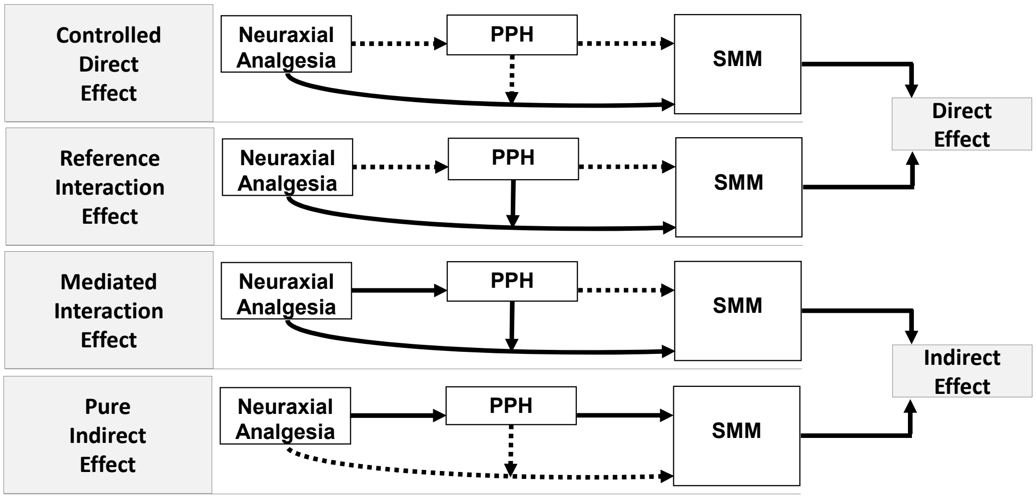

Solid lines indicate path for the effect of interest and dashed lines the effects held constant.

The component effects are estimated through logistic regression modeling. The model for estimating the component effects of the exposure (a) on the outcome (Y) in the presence of a potential moderator (m) with adjustment for other covariates (c) can be expressed as follows:

$$E\{P(Y=1|a,m,c)\} = \theta_0 + \theta_{1a} + \theta_{2m} + \theta_{3am} + \theta'_{4c} \quad (1)$$

$$E\{P(M=1|a,c)\} = \beta_0 + \beta_{1a} + \beta'_{2c} \quad (2)$$

Equation (1) models the effects of the exposure (a), moderator (m) and covariates (c) on the outcome (Y), where θ_0 denotes the intercept, θ_{1a} the exposure effect, θ_{2m} the moderator effect, θ_{3am} the interaction effect between the exposure and the moderator, and θ'_{4c} the effects of covariates. Equation (2) models the effect of the exposure and covariates on the moderator where β_0 denotes the intercept, β_{1a} the exposure effect on the moderator and β'_{2c} the effects of covariates on the moderator. If the exposure variable and the moderator variable are binary, the total effect of the exposure (a) on the outcome (Y) can be expressed as the sum of four components as follows:

Total effect of exposure=$(Y_{10} - Y_{00}) + (Y_{11} - Y_{10} - Y_{01} + Y_{00})(M_0) + (Y_{11} - Y_{10} - Y_{01} + Y_{00})(M_1 - M_0) + (Y_{01} - Y_{00})(M_1 - M_0)$, where $(Y_{10} - Y_{00})$ is the controlled direct effect, which is represented by θ_{1a}; $(Y_{11} - Y_{10} - Y_{01} + Y_{00})(M_0)$ is the reference interaction effect, which is equivalent to $\theta_{3am} \cdot \beta_0$; $(Y_{11} - Y_{10} - Y_{01} + Y_{00})(M_1 - M_0)$ is the mediated interaction effect, which is equivalent to $\theta_{3am} \bullet \beta_{1a}$; and $(Y_{01} - Y_{00})(M_1 - M_0)$ is the pure indirect effect, which is equivalent to $\theta_{2m} \bullet \beta_{1a}$. The NLMIXED procedure in SAS can be used to fit the above hierarchical model and estimate the component effects, and the delta method is used to compute 95% confidence intervals (Chihuri S and Li G "*Direct and indirect effects of marijuana use on the risk of fatal 2-vehicle crash initiation.*' Inj Epidemiol 2020;7(1): 49).

© 2022 Guglielminotti J et al. *JAMA Network Open*.

eFigure 3. Flowchart of the study

Abbreviations: AHA: American Hospital Association; FIPS: Federal Information Processing Standards; SID: state inpatient database; SMM: severe maternal morbidity
(a) Causes for exclusion are not mutually exclusive

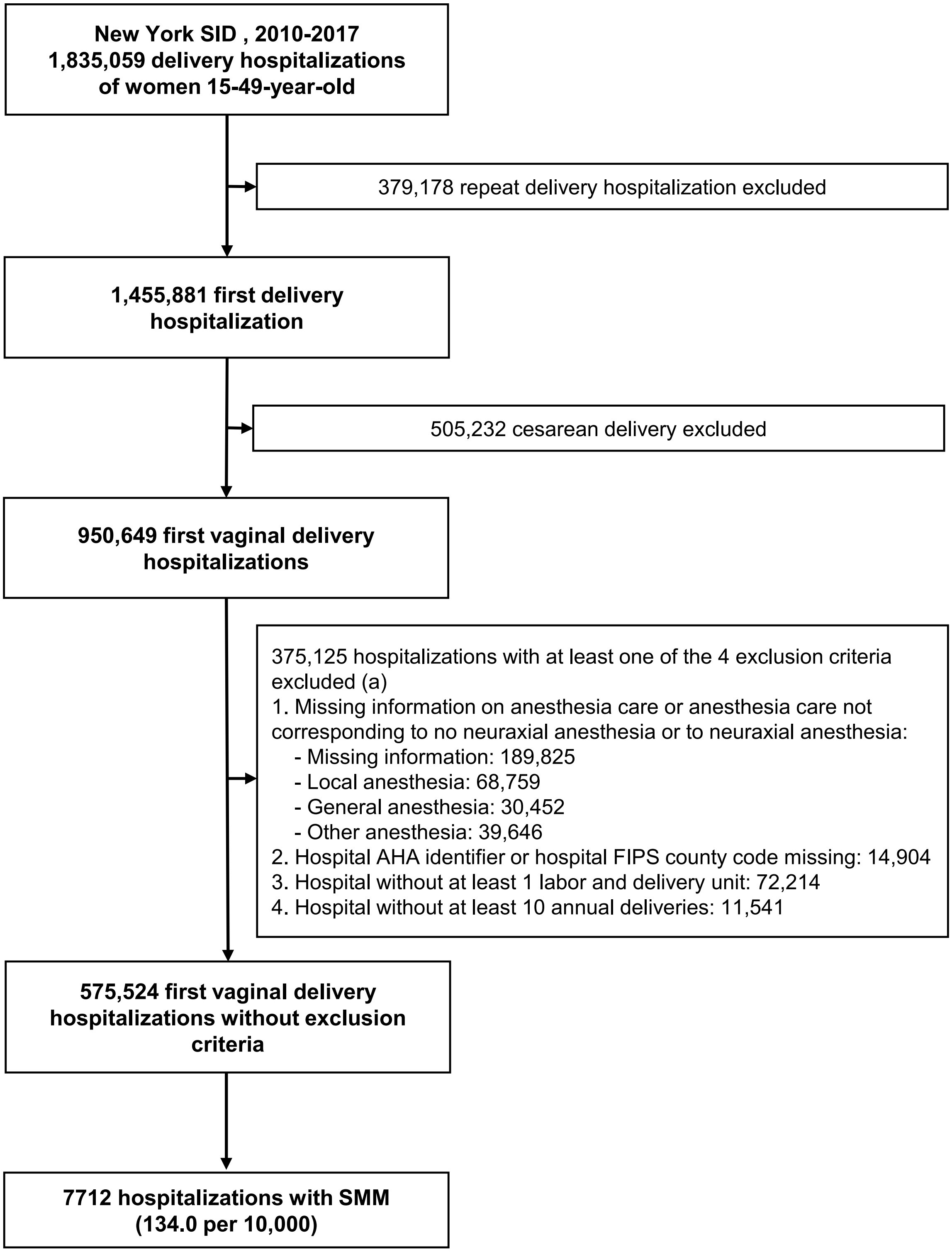

© 2022 Guglielminotti J et al. *JAMA Network Open*.

eTable 4. Comparison of women who did not receive neuraxial analgesia for vaginal delivery and those who did, before and after propensity-score matching (New York State hospitals, 2010-2017).

	**Before matching**			**After matching**		
	No neuraxial analgesia (n = 302,603)	Neuraxial analgesia (n = 272,921)	SMD	No neuraxial analgesia (n = 96,407)	Neuraxial analgesia (n = 96,407)	SMD
General characteristics						
Age (year)			7.3%			4.5%
≤ 19	21,983 (7.3%)	17,594 (6.4%)		6414 (6.7%)	7513 (7.8%)	
20-29	153,824 (50.8%)	131,552 (48.2%)		49,860 (51.7%)	49,226 (51.1%)	
30-39	117,305 (38.8%)	115,127 (42.2%)		37,106 (38.5%)	36,797 (38.2%)	
≥ 40	9491 (3.1%)	8648 (3.2%)		3027 (3.1%)	2871 (3.0%)	
Race (missing = 3206)			**30.8%**			<0.1%
Non-Hispanic White	118,533 (39.5%)	139,743 (51.4%)		45,562 (47.3%)	45,562 (47.3%)	
Non-Hispanic Black	54,749 (18.2%)	33,828 (12.4%)		13,861 (14.4%)	13,861 (14.4%)	
Hispanic	65,924 (22.0%)	38,942 (14.3%)		17,872 (18.5%)	17,872 (18.5%)	
Non-Hispanic Asian or Pacific Islander	20,796 (6.9%)	25,269 (9.3%)		6627 (6.9%)	6627 (6.9%)	
Other	40,219 (13.4%)	34,315 (12.6%)		12,485 (13.0%)	12,485 (13.0%)	
Rural residence (missing = 1761)	19,261 (6.4%)	12,517 (4.6%)	7.9%	6970 (7.2%)	6858 (7.1%)	<0.1%
Insurance type (missing = 3)			**39.0%**			6.9%
Medicaid	172,351 (57.0%)	111,516 (40.9%)		51,011 (52.9%)	50,013 (51.9%)	
Medicare	1382 (0.5%)	1101 (0.4%)		480 (0.5%)	452 (0.5%)	
Private	109,611 (36.2%)	149,732 (54.9%)		39,700 (41.2%)	40,412 (41.9%)	
Self-pay (uninsured)	11,354 (3.8%)	4494 (1.6%)		1690 (1.8%)	2533 (2.6%)	
Other	7902 (2.6%)	6078 (2.2%)		3526 (3.7%)	2997 (3.1%)	
CMI-OB ≥ 1	88,132 (29.1%)	87,046 (31.9%)	6.0%	28,732 (29.8%)	28,732 (29.8%)	<0.1%
Obesity	15,349 (5.1%)	13,579 (5.0%)	<1.0%	4216 (4.4%)	3468 (3.6%)	4.0%

© 2022 Guglielminotti J et al. *JAMA Network Open*.

eTable 4 (continued)

	**Before matching**			After matching		
	No neuraxial analgesia (n = 302,603)	Neuraxial analgesia (n = 272,921)	SMD	No neuraxial analgesia (n = 96,407)	Neuraxial analgesia (n = 96,407)	SMD
Possible contraindications to neuraxial anesthesia						
Coagulation factor deficit, Von Willebrand disease, and thrombocytopenia	5757 (1.9%)	6107 (2.2%)	2.4%	2003 (2.1%)	1851 (1.9%)	1.1%
Fever or infection during labor	2412 (0.8%)	2634 (1.0%)	1.8%	596 (0.6%)	558 (0.6%)	<0.1%
Chorioamnionitis	6044 (2.0%)	7622 (2.8%)	5.2%	1676 (1.7%)	1748 (1.8%)	<0.1%
Pregnancy and labor characteristics						
Admission for delivery during a weekend	72,604 (24.0%)	64,144 (23.5%)	1.2%	23,143 (24.0%)	22,835 (23.7%)	<0.1%
Pregnancy resulting from ART	553 (0.2%)	1148 (0.4%)	4.2%	233 (0.2%)	265 (0.3%)	<0.1%
Previous cesarean delivery	8257 (2.7%)	7476 (2.7%)	<1.0%	2692 (2.8%)	2977 (3.1%)	1.8%
Uterus fibroid	2392 (0.8%)	2485 (0.9%)	1.3%	705 (0.7%)	692 (0.7%)	<0.1%
Polyhydramnios	1911 (0.6%)	2308 (0.8%)	2.5%	663 (0.7%)	670 (0.7%)	<0.1%
Placenta praevia	401 (0.1%)	360 (0.1%)	<0.1%	145 (0.2%)	142 (0.1%)	<0.1%
Placenta accreta	913 (0.3%)	1171 (0.4%)	2.1%	354 (0.4%)	393 (0.4%)	<0.1%
Multiple gestation	2003 (0.7%)	2495 (0.9%)	2.9%	732 (0.8%)	843 (0.9%)	1.3%
Abnormal presentation	8999 (3.0%)	8062 (3.0%)	<1.0%	3118 (3.2%)	2984 (3.1%)	<0.1%
Preterm delivery	17,077 (5.6%)	12,705 (4.7%)	4.5%	5912 (6.1%)	5879 (6.1%)	<0.1%
Premature rupture of membranes	17,461 (5.8%)	24,265 (8.9%)	**12.0%**	6169 (6.4%)	5962 (6.2%)	<0.1%
Induction of labor	56,458 (18.7%)	67,178 (24.6%)	**14.5%**	19,887 (20.6%)	19,845 (20.6%)	<0.1%
Abnormal fetal heart rhythm	38,509 (12.7%)	50,405 (18.5%)	**15.9%**	11,964 (12.4%)	10,905 (11.3%)	3.4%

© 2022 Guglielminotti J et al. *JAMA Network Open*.

eTable 4 (continued)

	**Before matching**			**After matching**		
	No neuraxial analgesia (n = 302,603)	Neuraxial analgesia (n = 272,921)	SMD	No neuraxial analgesia (n = 96,407)	Neuraxial analgesia (n = 96,407)	SMD
Hospital characteristics						
Teaching hospital	240,277 (79.4%)	229,617 (84.1%)	**12.3%**	74,439 (77.2%)	74,926 (77.7%)	1.2%
Rural hospital	16,330 (5.4%)	7461 (2.7%)	**13.5%**	4965 (5.2%)	4532 (4.7%)	2.1%
Annual number of deliveries	2684 (1797)	3455 (2137)	**39.1%**	3018 (2241)	2999 (1995)	<0.1%
Cesarean delivery rate	33.3% (6.5)	34.4% (6.5)	**16.7%**	32.9% (7.1)	33.1% (6.8)	3.2%
Proportion of induction of labor	16.4% (7.6)	18.3% (7.8)	**24.9%**	17.7% (8.0)	17.4% (8.3)	3.9%
Proportion of racial and ethnic minority women	57.9% (31.2)	48.7% (26.3)	**32.2%**	49.2% (28.7)	48.0% (28.6)	4.1%
Proportion of safety net women (**a**)	58.2% (27.5)	43.5% (24.2)	**56.8%**	51.4% (23.6)	51.5% (23.9)	<0.1%
Proportion of women with CMI-OB ≥ 1	43.0% (6.5)	45.3% (7.1)	**33.8%**	43.0% (7.4)	42.2% (7.5)	**11.2%**
Proportion of admission for delivery during a weekend	20.7% (2.0)	20.5% (2.2)	7.4%	20.7% (2.2)	20.6% (2.1)	2.7%
Proportion of neuraxial techniques in deliveries	22.1% (28.0)	71.8% (17.3)	**213.3%**	57.7% (17.9)	58.7% (18.5)	5.4%
Coding intensity	8.0 (1.5)	8.3 (1.6)	**24.9%**	8.1 (1.6)	7.8 (1.6)	**17.9%**
Hospital county characteristics (per 1000 in-hospital births in the county)						
Number of obstetricians and gynecologists	13.4 (4.7)	14.7 (4.7)	**27.7%**	13.5 (5.3)	13.3 (4.9)	4.1%
Number of physician anesthesiologists	15.5 (6.9)	17.7 (6.2)	**33.8%**	16.0 (7.3)	15.6 (6.4)	5.5%
Number of certified registered nurse anesthetists	4.9 (4.4)	6.3 (5.5)	**27.9%**	5.9 (6.0)	5.6 (5.4)	5.4%
Other						
Year of delivery			5.1%			**66.7%**
2010-2011	91,737 (30.3%)	79,609 (29.2%)		29,327 (30.4%)	55,840 (57.9%)	
2012-2013	79,802 (26.4%)	69,220 (25.4%)		24,877 (25.8%)	23,706 (24.6%)	
2014-2015	75,845 (25.1%)	69,411 (25.4%)		24,472 (25.4%)	10,715 (11.1%)	
2016-2017	55,219 (18.2%)	54,681 (20.0%)		17,731 (18.4%)	6146 (6.4%)	

Abbreviations: ART: assisted reproductive technology; CMI-OB: comorbidity index for obstetric patients; SMD: standardized mean difference.
Footnote: Results expressed as count (%) or mean (1 standard deviation).
(**a**) Safety net women are Medicaid beneficiaries, Medicare beneficiaries, and uninsured

© 2022 Guglielminotti J et al. *JAMA Network Open*.

eTable 5. Odds ratios of postpartum hemorrhage associated with neuraxial analgesia for vaginal delivery with the successive addition of patient and hospital characteristics in the propensity score used for weighting (New York State hospitals, 2010-2017)

**No neuraxial analgesia**			**Neuraxial analgesia**				
No. of women	Nb. of events	Incidence (%; 95% CI)	No. of women	Nb. of events	Incidence (%; 95% CI)	Risk difference (%)	OR (95% CI)
Step 1:General characteristics (a)							
299,171	9674	3.23 (3.17, 3.30)	271,462	9334	3.44 (3.37, 3.51)	0.21	1.07 (1.04, 1.10)
Step 2:General characteristics and possible contraindications to neuraxial anesthesia (b)							
299,186	9769	3.27 (3.20, 3.33)	271,451	9255	3.41 (3.34, 3.48)	0.14	1.05 (1.02, 1.08)
Step 3: General characteristics, possible contraindications to neuraxial anesthesia, and labor characteristics (c)							
299,319	9859	3.29 (3.23, 3.36)	270,805	9128	3.37 (3.30, 3.44)	0.08	1.02 (0.99, 1.05)
Step 4: General characteristics, possible contraindications to neuraxial anesthesia, labor characteristics, and hospital characteristics (d)							
303,803	10823	3.56 (3.44, 3.68)	219,802	7265	3.31 (3.20, 3.41)	-0.25	**0.93 (0.90, 0.95)**
Step 5: General characteristics, possible contraindications to neuraxial anesthesia, labor characteristics, hospital characteristics, and hospital county characteristics (e)							
304,651	10791	3.54 (3.42, 3.66)	219,340	7279	3.32 (3.21, 3.43)	-0.22	**0.93 (0.91, 0.96)**

(**a**) General characteristics include: 1) age, 2) race and ethnicity, 3) health insurance type, 4) rural residence, 5) comorbidity index for obstetric patients, and 6) obesity.
(**b**) Possible contraindications to neuraxial anesthesia include: 1) coagulation factor deficit, Von Willebrand disease, and thrombocytopenia, 2) fever or infection during labor, and 3) chorioamnionitis.
(**c**) Labor characteristics include: 1) admission during a weekend, 2) pregnancy resulting from assisted reproductive technology, 3) previous cesarean delivery, 4) uterus fibroid, 5) polyhydramnios, 6) placenta praevia, 7) placenta accrete, 8) multiple gestation, 9) abnormal presentation, 10) preterm delivery, 11) premature rupture of membranes, 12) induction of labor, and 13) abnormal fetal heart rhythm.
(**d**) Hospital characteristics include: 1) teaching hospital, 2) rural hospital, 3) annual number of deliveries, 4) cesarean delivery rate, 5) proportion of induced of labor, 6) proportion of racial and ethnic minority women, 7) proportion of safety net women, 8) proportion of women with comorbidity index for obstetric patients ≥ 1, 9) proportion of admission for delivery during a weekend, 10) proportion of neuraxial analgesic or anesthetic techniques in deliveries, and 11) coding intensity.
(**e**) Hospital county characteristics include: 1) number of obstetricians and gynecologists, 2) number of physician anesthesiologists, and 3) number of certified registered nurse anesthetist.

© 2022 Guglielminotti J et al. *JAMA Network Open*.

eTable 6. Odds ratios of postpartum hemorrhage associated with neuraxial analgesia for vaginal delivery with the individual addition of each of the hospital characteristics to the propensity score used for weighting (New York State hospitals, 2010-2017)

In addition to each hospital characteristics, the propensity score include the 22 general characteristics, possible contraindications to neuraxial anesthesia, and labor characteristics reported in **eTable 3**.

**No neuraxial analgesia**			**Neuraxial analgesia**				
No. of women	Nb. of events	Incidence (%; 95% CI)	No. of women	Nb. of events	Incidence (%; 95% CI)	Risk difference (%)	OR (95% CI)
Proportion of neuraxial analgesic or anesthetic techniques in deliveries							
302,890	10,698	3.53 (3.42 ,3.65)	218,145	7271	3.33 (3.23 ,3.43)	-0.20	**0.94** (**0.91** ,**0.97**)
Coding intensity							
298,818	10,071	3.37 (3.30 ,3.44)	271,739	8947	3.29 (3.22 ,3.36)	-0.08	0.98 (0.95 ,1.00)
Proportion of women with a comorbidity index for obstetric patients ≥ 1							
298,606	10,073	3.37 (3.30 ,3.44)	272,091	8974	3.30 (3.23 ,3.37)	-0.07	0.98 (0.95 ,1.01)
Annual number of deliveries							
300,876	10,036	3.34 (3.27 ,3.41)	269,670	8972	3.33 (3.26 ,3.40)	-0.01	1.00 (0.97 ,1.03)
Proportion of induced labor							
298,781	9972	3.34 (3.27 ,3.41)	271,428	9032	3.33 (3.26 ,3.40)	-0.01	1.00 (0.97 ,1.03)
Proportion of safety net women							
299,942	9931	3.31 (3.24 ,3.38)	269,648	8994	3.34 (3.26 ,3.41)	0.03	1.01 (0.98 ,1.04)
Proportion of racial and ethnic minority women							
299,778	9954	3.32 (3.25 ,3.39)	270,304	9054	3.35 (3.28 ,3.42)	0.03	1.01 (0.98 ,1.04)
Teaching hospital							
298,988	9926	3.32 (3.25 ,3.39)	271,804	9095	3.35 (3.28 ,3.42)	0.03	1.01 (0.98 ,1.04)
Rural hospital							
299,072	9883	3.30 (3.24 ,3.37)	271,623	9129	3.36 (3.29 ,3.43)	0.06	1.02 (0.99 ,1.05)
Proportion of admission for delivery during a weekend							
299,185	9880	3.30 (3.24 ,3.37)	271,371	9136	3.37 (3.30 ,3.44)	0.07	1.02 (0.99 ,1.05)
Cesarean delivery rate							
299,633	9855	3.29 (3.22 ,3.36)	270,707	9126	3.37 (3.30 ,3.44)	0.08	**1.03 (1.00 ,1.06)**

© 2022 Guglielminotti J et al. *JAMA Network Open*.
